# Supplementary material for: Mortality Predictive Value of the C2HEST Score in Elderly Subjects with COVID-19—A Subanalysis of the COLOS Study
Source: J Clin Med. 2022 Feb 14;11(4):992. doi: 10.3390/jcm11040992 (PMC8879688; doi:10.3390/jcm11040992)
Supplement: Supplementary file 1 [file jcm-11-00992-s001.zip › jcm-1537834 supplementary.pdf]

Journal of Clinical Medicine

1. The strength of the association between CH2EST-score and study endpoints including non-fatal adverse events is presented in Figure S1 (OR overall and OR for low vs high C2HEST risk score) and Table S1

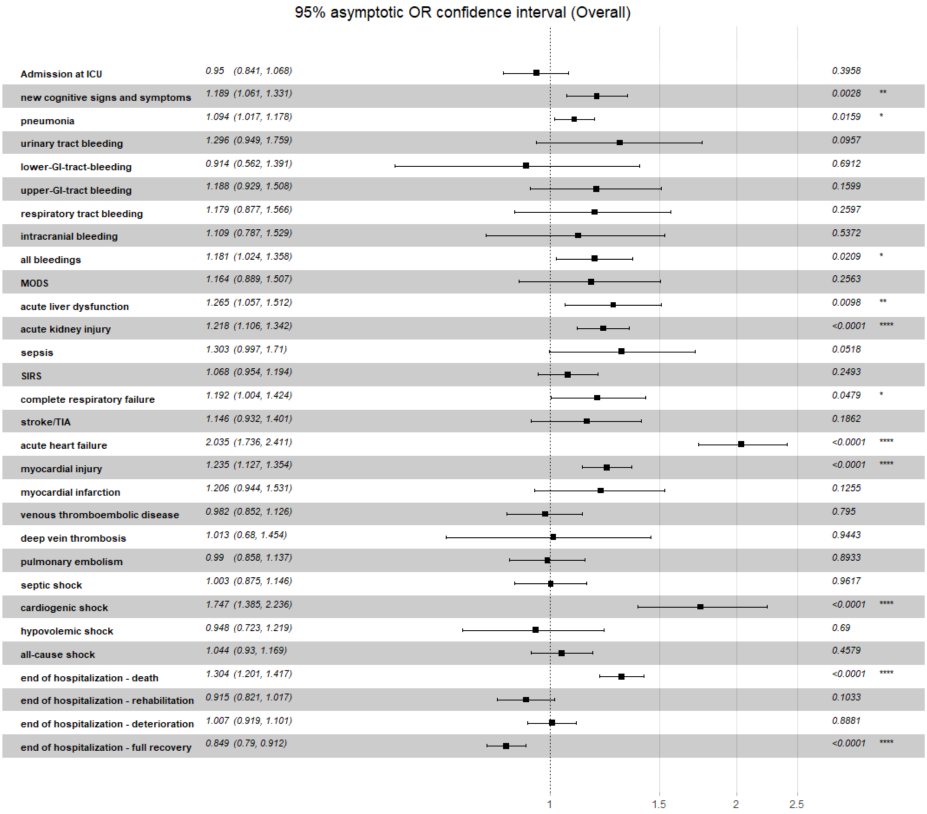

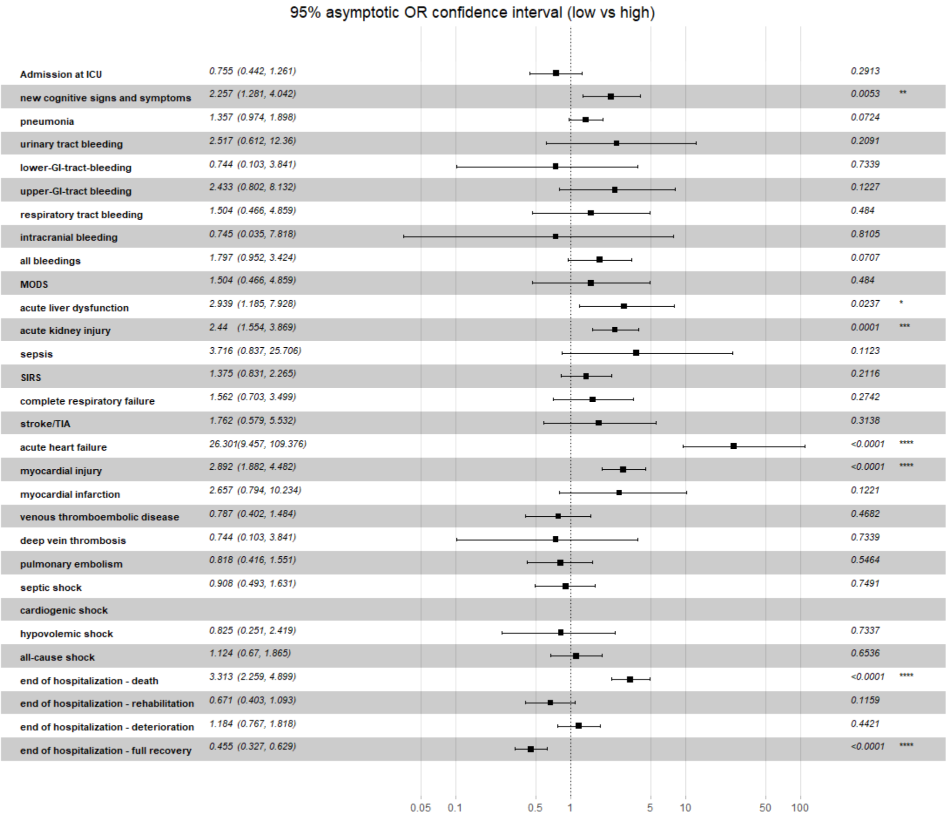

Figure S1. 95% Asymptotic OR Confidence Interval (low vs high).

Table S1. The strength of the association between CH2EST-score and study endpoints.

Endpoint	comparison	OR	Low CI	High CI	P value
End of hospitalization - full recovery	overall	0.849	0.790	0.912	< 0.0001
	low vs medium	0.573	0.432	0.758	0.0001
	low vs high	0.455	0.327	0.629	< 0.0001
End of hospitalization - deterioration	overall	1.007	0.919	1.101	0.8881
	low vs medium	1.467	1.018	2.126	0.0412
	low vs high	1.184	0.767	1.818	0.4421
End of hospitalization - rehabilitation	overall	0.915	0.821	1.017	0.1033
	low vs medium	0.882	0.589	1.320	0.5407
	low vs high	0.671	0.403	1.093	0.1159
End of hospitalization - death	overall	1.304	1.201	1.417	< 0.0001
	low vs medium	1.894	1.320	2.742	0.0006
	low vs high	3.313	2.259	4.899	< 0.0001
All-cause shock	overall	1.044	0.930	1.169	0.4579
	low vs medium	0.837	0.520	1.344	0.4609
	low vs high	1.124	0.670	1.865	0.6536
Hypovolemic shock	overall	0.948	0.723	1.219	0.69
	low vs medium	0.592	0.197	1.659	0.325
	low vs high	0.825	0.251	2.419	0.7337
Cardiogenic shock	overall	1.747	1.385	2.236	< 0.0001
	low vs medium	NA	NA	NA	NA
	low vs high	NA	NA	NA	NA
Septic shock	overall	1.003	0.875	1.146	0.9617
	low vs medium	0.706	0.406	1.217	0.212
	low vs high	0.908	0.493	1.631	0.7491

Endpoint	comparison	OR	Low CI	High CI	P value
pulmonary embolism	overall	0.990	0.858	1.137	0.8933
	low vs medium	0.890	0.511	1.552	0.6803
	low vs high	0.818	0.416	1.551	0.5464
deep vein thrombosis	overall	1.013	0.680	1.454	0.9443
	low vs medium	0.671	0.131	3.061	0.6025
	low vs high	0.744	0.103	3.841	0.7339
venous thromboembolic disease	overall	0.982	0.852	1.126	0.795
	low vs medium	0.890	0.516	1.537	0.6743
	low vs high	0.787	0.402	1.484	0.4682
myocardial infarction	overall	1.206	0.944	1.531	0.1255
	low vs medium	2.041	0.659	7.585	0.2383
	low vs high	2.657	0.794	10.234	0.1221
myocardial injury	overall	1.235	1.127	1.354	< 0.0001
	low vs medium	1.790	1.198	2.697	0.0048
	low vs high	2.892	1.882	4.482	< 0.0001
acute heart failure	overall	2.035	1.736	2.411	< 0.0001
	low vs medium	4.298	1.390	18.751	0.0228
	low vs high	26.301	9.457	109.376	< 0.0001
stroke/TIA	overall	1.146	0.932	1.401	0.1862
	low vs medium	2.448	0.995	6.884	0.0643
	low vs high	1.762	0.579	5.532	0.3138
complete respiratory failure	overall	1.192	1.004	1.424	0.0479
	low vs medium	1.123	0.528	2.391	0.7626
	low vs high	1.562	0.703	3.499	0.2742
SIRS	overall	1.068	0.954	1.194	0.2493
	low vs medium	0.913	0.566	1.472	0.7075
	low vs high	1.375	0.831	2.265	0.2116
sepsis	overall	1.303	0.997	1.710	0.0518
	low vs medium	3.236	0.766	21.976	0.1474
	low vs high	3.716	0.837	25.706	0.1123
acute kidney injury	overall	1.218	1.106	1.342	< 0.0001
	low vs medium	1.502	0.974	2.341	0.0682
	low vs high	2.440	1.554	3.869	0.0001
acute liver dysfunction	overall	1.265	1.057	1.512	0.0098
	low vs medium	2.199	0.937	5.746	0.0834
	low vs high	2.939	1.185	7.928	0.0237
MODS	overall	1.164	0.889	1.507	0.2563
	low vs medium	0.745	0.213	2.492	0.6289
	low vs high	1.504	0.466	4.859	0.484
all bleedings	overall	1.181	1.024	1.358	0.0209
	low vs medium	0.991	0.524	1.889	0.9788
	low vs high	1.797	0.952	3.424	0.0707
intracranial bleeding	overall	1.109	0.787	1.529	0.5372
	low vs medium	3.640	0.905	24.217	0.1036
	low vs high	0.745	0.035	7.818	0.8105
respiratory tract bleeding	overall	1.179	0.877	1.566	0.2597
	low vs medium	0.296	0.043	1.293	0.1372
	low vs high	1.504	0.466	4.859	0.484
upper-GI-tract bleeding	overall	1.188	0.929	1.508	0.1599
	low vs medium	1.261	0.399	4.291	0.6945
	low vs high	2.433	0.802	8.132	0.1227

Endpoint	comparison	OR	Low CI	High CI	P value
lower-GI-tract-bleeding	overall	0.914	0.562	1.391	0.6912
	low vs medium	0.222	0.011	1.512	0.1797
	low vs high	0.744	0.103	3.841	0.7339
urinary tract bleeding	overall	1.296	0.949	1.759	0.0957
	low vs medium	1.198	0.263	6.117	0.8135
	low vs high	2.517	0.612	12.360	0.2091
pneumonia	overall	1.094	1.017	1.178	0.0159
	low vs medium	1.156	0.868	1.539	0.3211
	low vs high	1.357	0.974	1.898	0.0724

2. C2HEST-OD – expansion of the C2HEST score by adding the presence of diabetes mellitus and obesity

In order to verify if including obesisty and diabetes mellitus to the extended version of the C2HEST score (C2HEST-OD), we have performed the analysis presented below:

A.1 All-cause mortality (Table S2)

Table S2. All-cause mortality.

	Hazard ratio	Lower CI limit	Upper CI limit	p-value
Overall	1.2382	1.1335	1.3525	<0.0001
low vs medium	2.6670	1.3568	5.2422	0.0044
low vs high	4.2077	2.2037	8.0340	<0.0001

The change from the low to the high risk group increased the risk for death 4.21-fold vs 2.70 fold in the original version of the scale.

A.2 In-hospital mortality(Table S3)

Table S3. In-hospital mortality.

	Hazard ratio	Lower CI limit	Upper CI limit	p-value
Overall	1.2052	1.0821	1.3424	0.0007
low vs medium	1.7960	0.8411	3.8348	0.1303
low vs high	2.8402	1.3798	5.8462	0.0046

A.3. Clinical non-fatal events

The strength of the association between CH2EST-OD score and study endpoints including non-fatal adverse events is presented in supplementary Table 4

Table S4. The strength of the association between CH2EST-score and study endpoints.

Endpoint	Type	Odds.ratio	Lower.CI.limit	Upper.CI.limit	p.value
end of hospitalization - full recovery	Overall	0.749	0.642	0.865	0.0001
	low vs medium	0.421	0.195	0.884	0.0244
	low vs high	0.219	0.101	0.456	<0.0001
end of hospitalization - deterioration	Overall	1.044	0.798	1.343	0.7451
	low vs medium	1.791	0.369	12.871	0.4968
	low vs high	2.154	0.496	14.858	0.3508
end of hospitalization - rehabilitation	Overall	1.059	0.854	1.303	0.5888
	low vs medium	0.969	0.291	3.454	0.9596
	low vs high	1.200	0.399	4.057	0.7529
end of hospitalization - death	Overall	1.290	1.120	1.497	0.0005
	low vs medium	2.575	1.113	6.402	0.0325
	low vs high	4.245	1.901	10.304	0.0007
all-cause shock	Overall	1.006	0.864	1.168	0.9337
	low vs medium	1.760	0.763	4.281	0.1952
	low vs high	1.397	0.611	3.367	0.4388
hypovolemic shock	Overall	0.668	0.403	1.000	0.0769
	low vs medium	1.169	0.273	5.922	0.8359
	low vs high	NA	NA	NA	NA
cardiogenic shock	Overall	1.726	1.183	2.664	0.0069
	low vs medium	NA	NA	NA	NA
	low vs high	NA	NA	NA	NA
septic shock	Overall	1.057	0.902	1.235	0.4897
	low vs medium	1.899	0.746	5.289	0.1935
	low vs high	2.145	0.877	5.826	0.1095
pulmonary embolism	Overall	0.798	0.571	1.068	0.1551
	low vs medium	1.958	0.534	9.300	0.3396
	low vs high	0.378	0.048	2.355	0.2954
deep vein thrombosis	Overall	0.957	0.273	2.376	0.9291
	low vs medium	NA	NA	NA	NA
	low vs high	NA	NA	NA	NA
venous thromboembolic disease	Overall	0.798	0.571	1.068	0.1551
	low vs medium	1.958	0.534	9.300	0.3396
	low vs high	0.378	0.048	2.355	0.2954

Endpoint	Type	Odds.ratio	Lower.CI.limit	Upper.CI.limit	p.value
myocardial infarction	Overall	1.202	0.842	1.694	0.2929
	low vs medium	0.338	0.015	3.623	0.3813
	low vs high	1.185	0.223	8.779	0.8478
myocardial injury	Overall	1.169	0.991	1.389	0.0678
	low vs medium	2.059	0.758	6.063	0.1687
	low vs high	3.217	1.216	9.309	0.0229
acute heart failure	Overall	2.699	1.874	4.315	<0.0001
	low vs medium	NA	NA	NA	NA
	low vs high	NA	NA	NA	NA
stroke/TIA	Overall	0.413	0.025	1.458	0.3357
	low vs medium	NA	NA	NA	NA
	low vs high	NA	NA	NA	NA
complete respiratory failure	Overall	1.054	0.784	1.433	0.7266
	low vs medium	0.343	0.038	2.267	0.2867
	low vs high	0.550	0.067	3.319	0.5322
SIRS	Overall	1.072	0.865	1.318	0.5163
	low vs medium	1.238	0.353	4.955	0.7443
	low vs high	1.709	0.548	6.456	0.382
sepsis	Overall	1.155	0.593	2.164	0.6393
	low vs medium	NA	NA	NA	NA
	low vs high	NA	NA	NA	NA
acute kidney injury	Overall	1.179	1.015	1.373	0.0315
	low vs medium	1.899	0.746	5.289	0.1935
	low vs high	3.181	1.332	8.517	0.0133
acute liver dysfunction	Overall	1.294	0.965	1.738	0.081
	low vs medium	2.104	0.260	43.258	0.5249
	low vs high	3.711	0.608	71.237	0.2315
MODS	Overall	1.092	0.787	1.483	0.5819
	low vs medium	2.130	0.264	43.765	0.5179
	low vs high	3.062	0.476	59.595	0.3134
all bleedings	Overall	1.058	0.830	1.334	0.6352
	low vs medium	3.871	0.963	25.955	0.0898
	low vs high	1.500	0.310	10.758	0.6358
intracranial bleeding	Overall	0.837	0.334	1.633	0.6408
	low vs medium	NA	NA	NA	NA
	low vs high	NA	NA	NA	NA
respiratory tract bleeding	Overall	1.244	0.851	1.802	0.2441
	low vs medium	NA	NA	NA	NA
	low vs high	NA	NA	NA	NA
upper-GI-tract bleeding	Overall	1.036	0.683	1.505	0.8572
	low vs medium	2.130	0.264	43.765	0.5179
	low vs high	1.181	0.110	25.795	0.8932
lower-GI-tract-bleeding	Overall	0.957	0.273	2.376	0.9291
	low vs medium	NA	NA	NA	NA

Endpoint	Type	Odds.ratio	Lower.CI.limit	Upper.CI.limit	p.value
	low vs high	NA	NA	NA	NA
urinary tract bleeding	Overall	0.710	0.243	1.456	0.4264
	low vs medium	0.690	0.027	17.731	0.7949
	low vs high	NA	NA	NA	NA
pneumonia	Overall	1.058	0.920	1.220	0.4353
	low vs medium	0.938	0.440	1.978	0.8659
	low vs high	1.276	0.605	2.670	0.5175
new cognitive signs and symptoms	Overall	0.990	0.667	1.407	0.9577
	low vs medium	NA	NA	NA	NA
	low vs high	0.774	0.164	4.067	0.7439
Admission at ICU	Overall	0.931	0.806	1.071	0.3214
	low vs medium	1.097	0.516	2.363	0.8103
	low vs high	0.954	0.457	2.019	0.8997

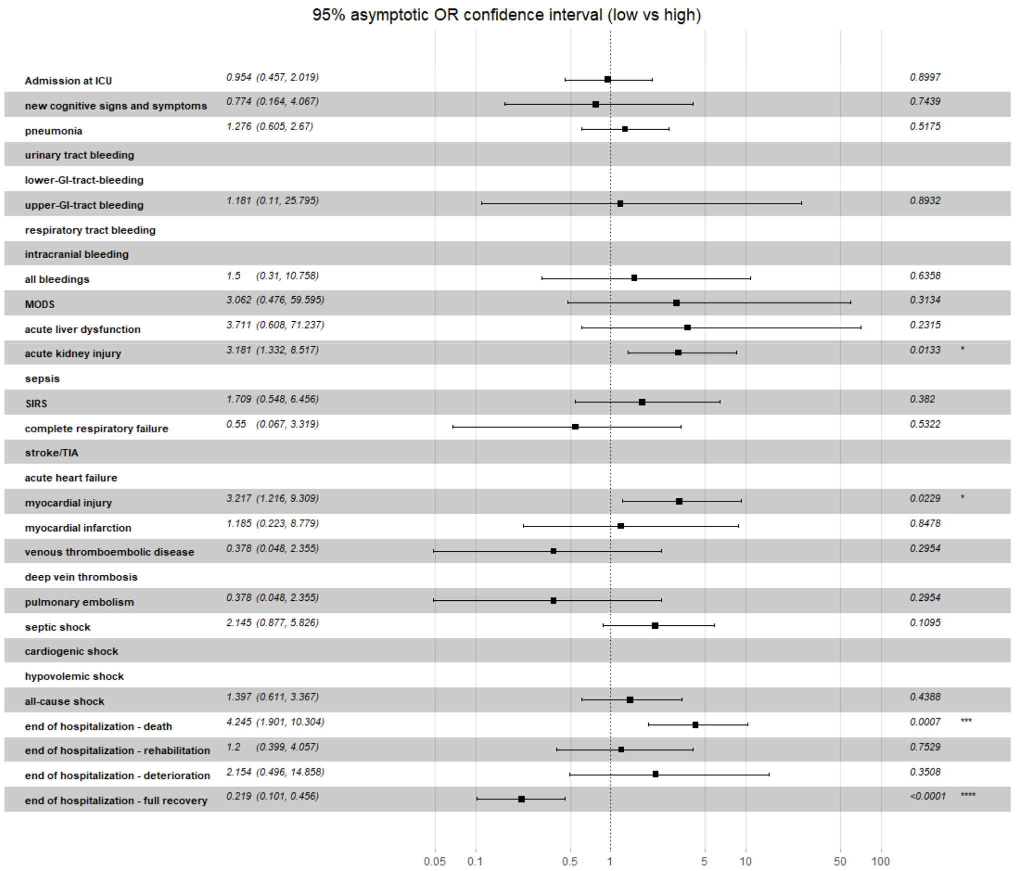

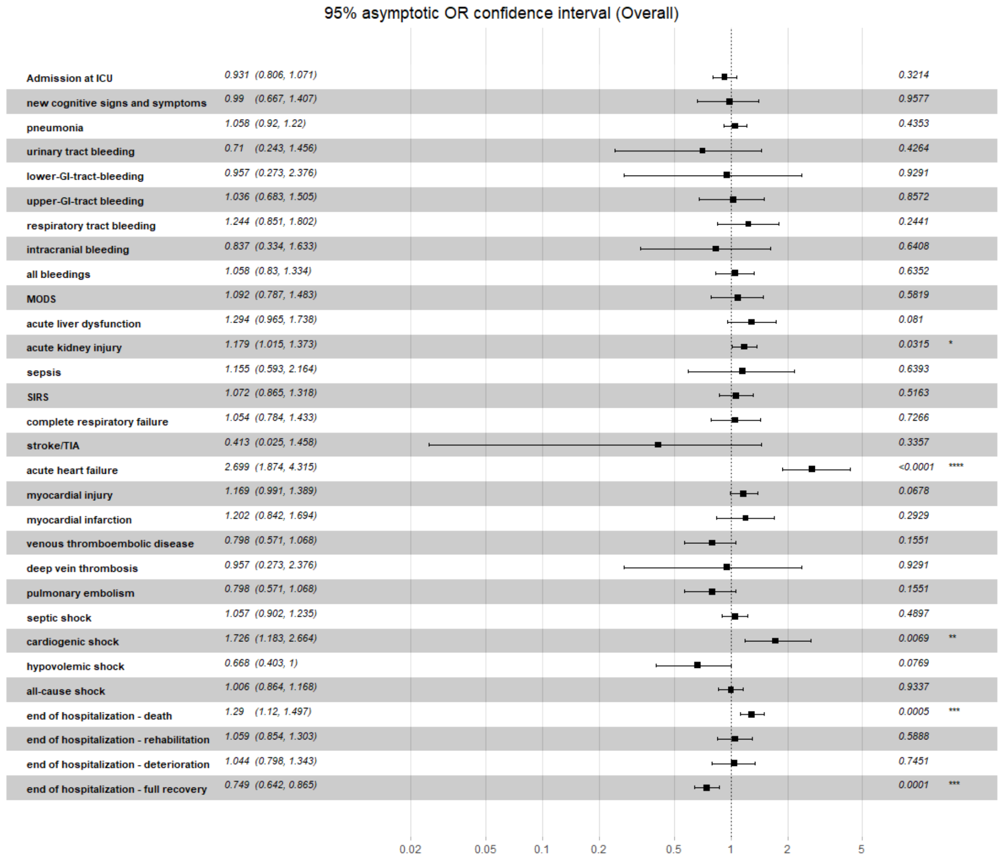

Figure S2. 95% Asymptotic OR Confidence Interval (low vs high & Overall).

3. Sub-analysis of the survival based on the ICU admission in particular C2HEST score strata

The presented below Kaplan-Meier (Figure S3)survival function presents the usefulness of the C2HEST score in predicting the mortality of elderly subjects who were admitted directly to the intensive care unit (due to COVID severity) vs. those admitted to the non-intensive ward of the medical university center due to COVID-19. The C2HEST score revealed to determine the outcome irrespectively on the initial symptom severity with the log-rank p-value of <0.0001:

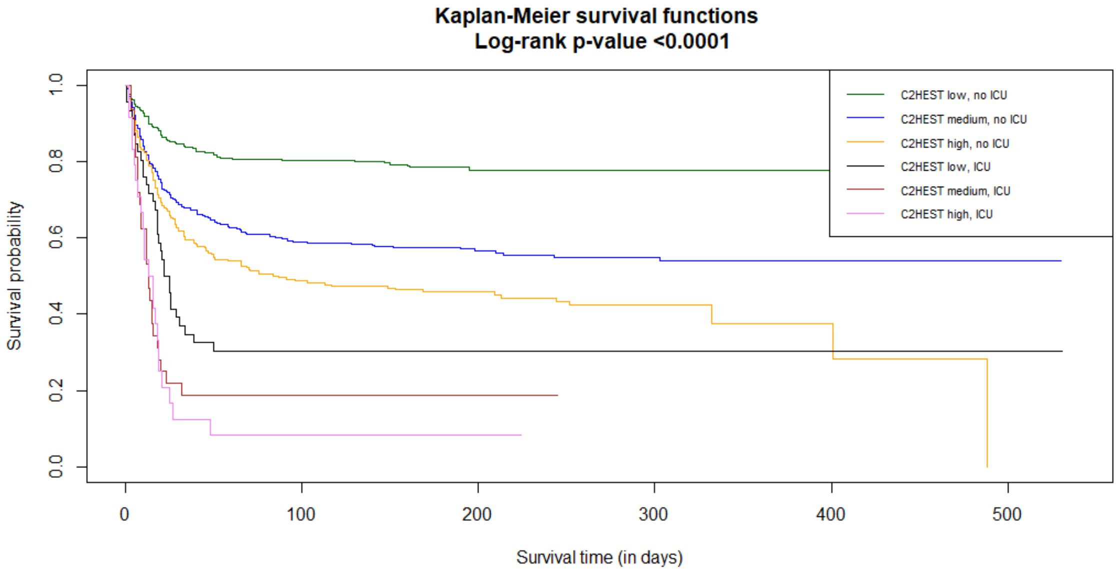

Figure S3. Kaplan-Meier survival function.

The C2HEST predictive value in transfer of COVID-19 elderly subjects to the ICU following clinical deterioration is presented in the Table S5:

Table S5. The C2HEST predictive value in transfer of COVID-19 elderly subjects to the ICU following clinical deterioration.

Endpoint	Type	Odds.ratio	Lower.CI.limit	Upper.CI.limit	p.value
Admission at ICU	Overall	0.950	0.841	1.068	0.3958
	low vs medium	0.593	0.366	0.950	0.031
	low vs high	0.755	0.442	1.261	0.2913

Also, the logistic regression model was developed to analyze the description of the admission at ICU by particular C2HEST components, where the strongest predictive value for ICU admission had the presence of hypertension, coronary artery disease and HFrEF (Table S6).

Table S6. The logistic regression model.

Endpoint	Component	Odds.ratio	Lower.CI.limit	Upper.CI.limit	p.value
Admission at ICU	Coronary artery disease	1.443	0.831	2.446	0.1814
	COPD	0.631	0.212	1.508	0.3476
	Age	0.351	0.218	0.549	<0.0001
	Thyroid Disease	0.373	0.142	0.810	0.0239
	Hypertension	1.794	1.092	3.051	0.0251
	HFrEF	1.519	0.879	2.574	0.1261
